# Case Report: Synchronous Esophageal Squamous Cell Carcinoma and Gastric Cardia Adenocarcinoma with Hepatoid Differentiation After Neoadjuvant Chemoimmunotherapy

**DOI:** 10.3390/jcm15155979

**Published:** 2026-07-31

**Authors:** Chengang Weng, Qing Yang, Xinlei Cao, Feng Gao

**Affiliations:** 1 Department of Thoracic Surgery, The Fourth Hospital of Hebei Medical University, Hebei Medical University, Shijiazhuang 050011, China; xwwcg0019@163.com (C.W.); 15130150603@163.com (X.C.); 2 Department of Laboratory Medicine, The Fourth Hospital of Hebei Medical University, Hebei Medical University, Shijiazhuang 050011, China; 18733156155@163.com

**Keywords:** hepatoid adenocarcinoma, gastric cardia, esophageal squamous cell carcinoma, alpha-fetoprotein, chemoimmunotherapy, tumor regression, case report

## Abstract

**Background/Objectives:** Hepatoid adenocarcinoma is a rare adenocarcinoma subtype. Synchronous esophageal squamous cell carcinoma (ESCC) and gastric cardia adenocarcinoma with hepatoid differentiation is exceptionally uncommon. This report describes lesion-specific staging, pathological response assessment, and individualized management after neoadjuvant chemoimmunotherapy. **Methods:** De-identified clinical, imaging, laboratory, operative, and pathological data were retrospectively reviewed. Both lesions were staged according to the AJCC 8th edition and reassessed using the CAP modified Ryan four-tier tumor regression grading system. **Results:** A 61-year-old man presenting with dysphagia had separate ESCC and gastric cardia adenocarcinoma, both staged cT2N0M0. One cycle of pembrolizumab 200 mg, nab-paclitaxel 300 mg, and cisplatin 100 mg was administered. Agranulocytosis precluded further neoadjuvant therapy; after neutrophil recovery, R0 resection was performed. Pathological review showed ESCC ypT1aN0M0, score 1, and cardia adenocarcinoma ypT1aN0M0, score 3, with approximately 30% regional hepatoid differentiation. All 10 examined lymph nodes were negative. AFP was not measured before treatment; post-treatment/preoperative values were 14.45 and 14.68 ng/mL, and the postoperative value was 6.08 ng/mL. Postoperative tislelizumab monotherapy was selected as an individualized, non-standard strategy, mainly because of affordability. At approximately 6 months after surgery, no recurrence, metastasis, or maintenance-related adverse event was identified. **Conclusions:** This case emphasizes biopsy under-sampling and lesion-specific staging and pathological assessment. The regression-score difference is descriptive and confounded by baseline burden, histology, and one-cycle exposure; the AFP findings do not validate a surveillance biomarker.

## 1. Introduction

Hepatoid adenocarcinoma (HAC) is a rare adenocarcinoma subtype characterized by morphological or immunophenotypic resemblance to hepatocellular carcinoma. The stomach is the most frequently reported primary site, and gastric HAC is associated with lymphovascular invasion, lymph node metastasis, liver metastasis, and poor prognosis [1,2,3,4,5]. Diagnosis requires integration of clinical, radiological, morphological, and immunohistochemical findings because HAC may mimic hepatocellular carcinoma and can be highly heterogeneous [3,6,7,8].

HAC arising in the gastric cardia without hepatic disease is uncommon, and coexistence with a separate primary ESCC is exceptionally rare. Small endoscopic biopsies may detect conventional adenocarcinoma but miss a geographically limited hepatoid component. Comprehensive examination of the resection specimen is therefore important when morphology, biomarkers, or clinical behavior are discordant.

Immune-checkpoint inhibitors combined with chemotherapy have demonstrated activity in advanced ESCC and gastric or gastroesophageal-junction adenocarcinoma [9,10,11,12]. MATTERHORN evaluated perioperative durvalumab plus FLOT specifically in resectable gastric or gastroesophageal-junction adenocarcinoma and therefore did not provide a unified regimen for a patient with synchronous ESCC [13]. Evidence for hepatoid adenocarcinoma remains limited to case reports and retrospective series [14,15,16]. The educational value of this case lies primarily in the rare synchronous presentation, the diagnostic limitations of small biopsies, and the need to assess each lesion separately after systemic treatment.

This report provides AJCC 8th-edition staging for both lesions, an explicit pathological regression method, the exact neoadjuvant exposure and adverse events, the rationale for treatment selection, and a defined follow-up interval. Future research should include standardized pathological review, paired molecular and immune profiling of synchronous lesions, and longer prospective follow-up.

## 2. Materials and Methods

### 2.1. Case Data Sources and Clinical Review

Clinical information was retrospectively extracted from de-identified inpatient and outpatient records, endoscopy reports, radiology reports, laboratory results, operative records, and the final pathology report. The chronology, drug doses, adverse events, and follow-up status were cross-checked against the source record by the clinical authors. Imaging was reviewed for local extent, nodal findings, and distant metastasis. No experimental intervention or research-specific assay was performed for this report.

### 2.2. Staging and Pathological Response Assessment

Clinical and pathological stages were assigned according to the AJCC Cancer Staging Manual, 8th edition [17]. The baseline stages determined by multidisciplinary review were ESCC cT2N0M0 (clinical stage II) and gastric cardia adenocarcinoma cT2N0M0 (clinical stage I). Because the cardia tumor epicenter did not involve the esophagogastric junction, gastric rather than esophageal/EGJ staging was applied. The resection specimens were staged as ESCC ypT1aN0M0 and gastric cardia adenocarcinoma ypT1aN0M0. Both lesions were retrospectively reviewed by the pathologist using the CAP modified Ryan four-tier tumor regression grading system, in which score 0 indicates complete response, score 1 near-complete response, score 2 partial response, and score 3 poor or no response [18,19].

### 2.3. Use of Generative Artificial Intelligence

ChatGPT (OpenAI OpCo, LLC, San Francisco, CA, USA; GPT-5.6 Thinking; https://chatgpt.com/; accessed on 19 July 2026) was used during manuscript revision to assist with English-language editing, structural organization, and drafting of the point-by-point response. The tool was not used to generate or alter clinical data, images, staging assignments, pathological diagnoses, laboratory results, or references. All AI-assisted text was independently checked against the source medical record and the primary literature by the authors, who take full responsibility for the manuscript.

## 3. Detailed Case Description

### 3.1. Patient Information

A 61-year-old man presented with a 3-month history of chest and back discomfort accompanied by mild postprandial dysphagia, particularly with solid food. He had no known history of hypertension, diabetes mellitus, coronary heart disease, viral hepatitis, tuberculosis, previous surgery, blood transfusion, or drug allergy. He had smoked 20 cigarettes per day for 40 years (approximately 40 pack-years) and had consumed approximately 300 mL of alcoholic beverages per week for 40 years. No relevant family history of malignancy was reported. Physical examination was unremarkable.

### 3.2. Clinical Findings and Timeline

Initial endoscopy showed two separate ulcerative, protruding, friable lesions: one in the esophagus approximately 30–35 cm from the incisors and one below the dentate line in the gastric cardia. A small nodular elevation near the gastric body junction showed chronic inflammation and mild intestinal metaplasia. The clinical course is summarized in Table 1.

### 3.3. Diagnostic Assessment and Clinical Staging

**Figure 1:**
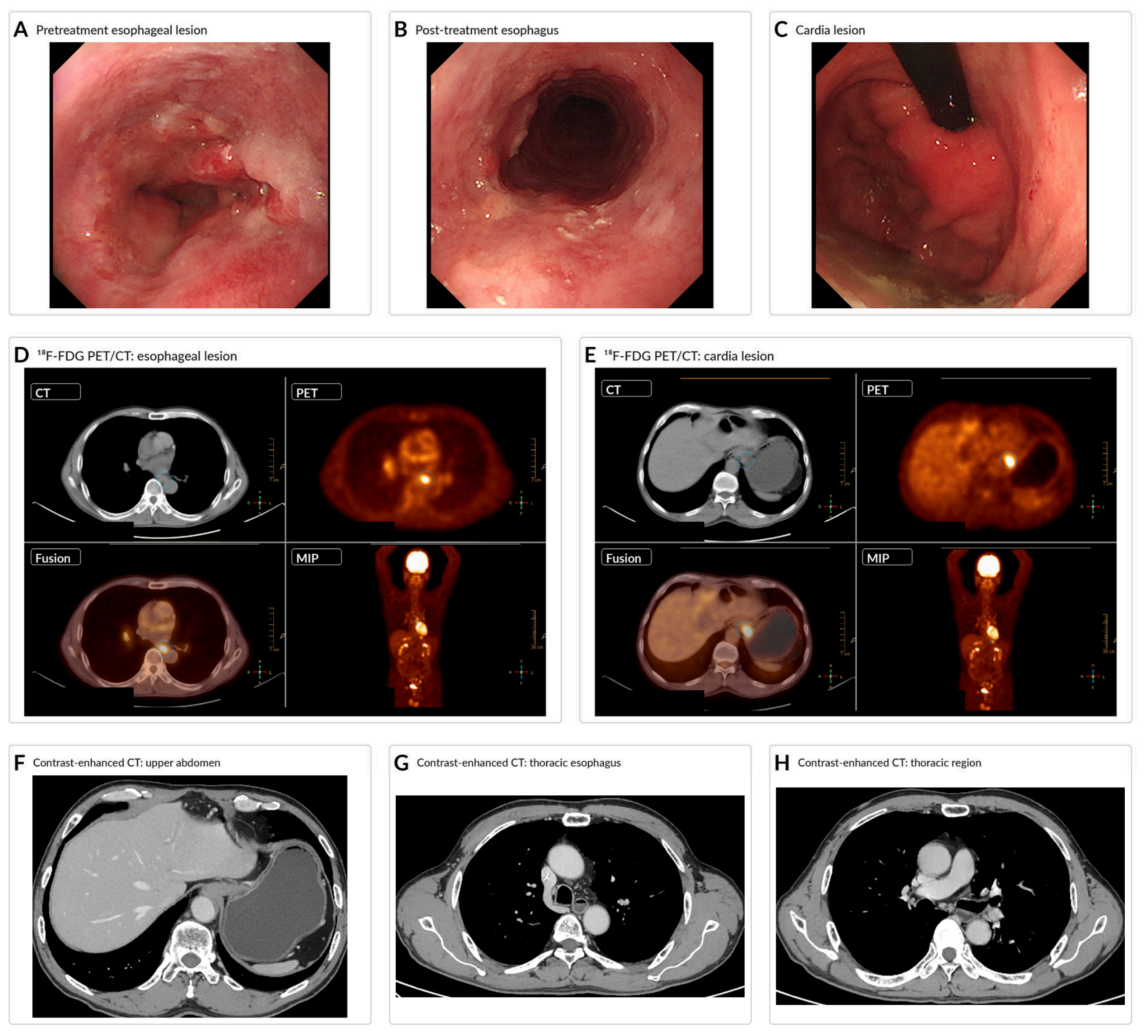
Endoscopic and radiological findings before and after treatment. (**A**) Pretreatment endoscopic appearance of the thoracic esophageal lesion. (**B**) Restaging endoscopy after one cycle of neoadjuvant chemoimmunotherapy, showing improvement of the esophageal lumen with residual mucosal abnormality. (**C**) Endoscopic appearance of the gastric cardia lesion. (**D**) Representative 18F-FDG PET/CT images of the esophageal lesion, including axial CT, axial PET, fused PET/CT, and maximum-intensity-projection views. (**E**) Representative 18F-FDG PET/CT images of the gastric cardia lesion in the corresponding four views. (**F**) Contrast-enhanced CT of the upper abdomen showing the cardia region. (**G**,**H**) Contrast-enhanced CT images of the thoracic esophagus and chest obtained for staging. No definite distant metastasis was identified.

Endoscopic biopsies showed ESCC and gastric cardia adenocarcinoma. Baseline CEA, CA19-9, and CA72-4 were not markedly elevated. Hepatitis B and C testing did not suggest active infection, and liver function was largely normal. PET/CT showed FDG uptake in the middle-to-lower thoracic esophageal lesion (SUVmax approximately 7.8) and gastric cardia lesion (SUVmax approximately 6.5), without distant metastasis (Figure 1). Multidisciplinary review assigned AJCC 8th-edition stages of cT2N0M0 (clinical stage II) for the ESCC and cT2N0M0 (clinical stage I, gastric staging) for the cardia adenocarcinoma. The cardia lesion was considered resectable because there was no unresectable local extension or distant disease, and its epicenter did not involve the esophagogastric junction.

The differential diagnosis of the cardia lesion included conventional adenocarcinoma, adenocarcinoma with hepatoid differentiation, metastatic hepatocellular carcinoma, and mixed histology. The pretreatment biopsy identified adenocarcinoma but did not capture the hepatoid component. The absence of radiological liver cancer or liver metastasis and the resection-specimen morphology and immunophenotype supported a primary gastric cardia origin.

### 3.4. Therapeutic Intervention

After multidisciplinary discussion, the team administered one cycle of pembrolizumab 200 mg, nab-paclitaxel 300 mg, and cisplatin 100 mg in December 2025. The immunotherapy-taxane-platinum approach was selected primarily to address the symptomatic ESCC while providing cytotoxic coverage for both upper gastrointestinal tumors. MATTERHORN durvalumab-FLOT was not selected because that regimen was studied specifically in resectable gastric or gastroesophageal-junction adenocarcinoma and did not address synchronous ESCC [13]. Accordingly, the chosen regimen was an individualized multidisciplinary strategy rather than a standardized perioperative protocol for both histologies.

During the second hospitalization, the patient developed agranulocytosis, and the planned subsequent cycle of chemotherapy and immunotherapy was withheld. Liver and renal function remained stable, and no overt immune-related endocrine or cardiac toxicity was detected. After the neutrophil count recovered, the multidisciplinary team recommended proceeding directly to surgery to avoid further treatment-related toxicity and delay. Restaging endoscopy and CT showed improvement of the esophageal lesion but persistent cardia wall thickening and enhancement. The patient underwent R0 resection on 21 January 2026; estimated blood loss was approximately 50 mL, and no transfusion was required.

### 3.5. Postoperative Pathology

**Figure 2:**
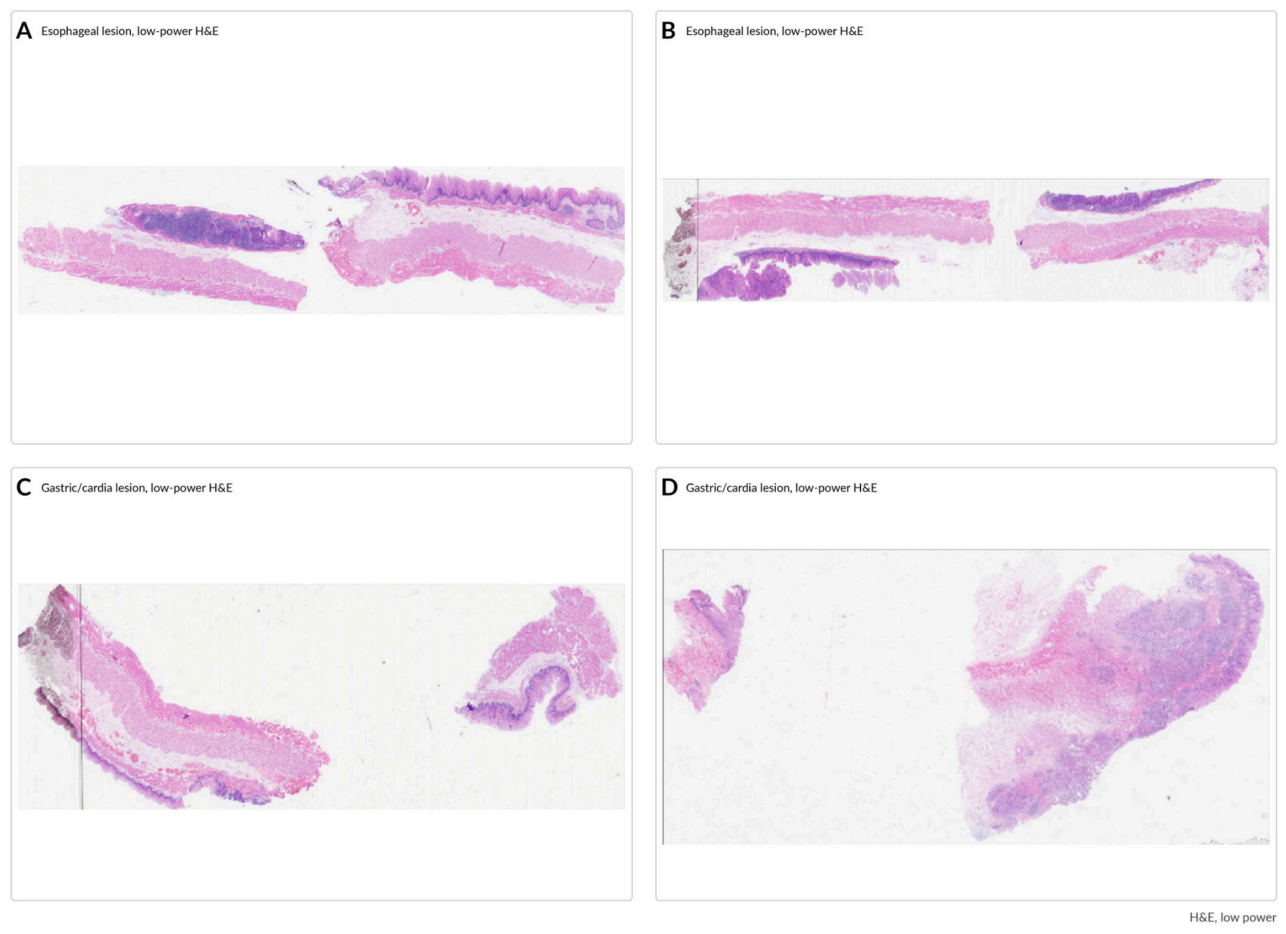
Low-power hematoxylin and eosin (H&E) findings of the resected lesions. (**A**,**B**) Representative low-power H&E sections of the esophageal lesion after neoadjuvant treatment, showing high-grade squamous intraepithelial neoplasia with focal carcinoma invading the lamina propria. (**C**,**D**) Representative low-power H&E sections of the gastric cardia lesion, showing residual adenocarcinoma with regional hepatoid differentiation and invasion of the muscularis mucosae. The panels show low-power morphology only; the supporting immunohistochemical findings are reported in the main text and Supplementary Table S1.

The esophageal lesion showed high-grade squamous intraepithelial neoplasia with focal carcinoma invading the lamina propria, no lymphovascular or perineural invasion, and negative margins; the pathological stage was ypT1aN0M0. The cardia lesion, whose epicenter did not involve the esophagogastric junction, showed grade II–III adenocarcinoma, Lauren mixed intestinal-diffuse type, with approximately 30% regional hepatoid differentiation and invasion of the muscularis mucosae; the pathological stage was ypT1aN0M0. Immunohistochemistry showed AE1/AE3 positivity, partial CDX2 positivity, and regional Glypican-3 and HepPar-1/Hep-1 positivity; CEA, synaptophysin, and P40 were negative. Both lesions were retrospectively reviewed by the pathologist using the CAP modified Ryan four-tier system: score 1 for the esophageal lesion and score 3 for the cardia lesion. No metastasis was identified in five paraesophageal or five cardia lymph nodes. The limited total yield of 10 nodes reduces confidence in the ypN0 designation and is acknowledged as a limitation (Figure 2).

### 3.6. AFP Measurements, Postoperative Treatment, and Follow-Up

AFP was not measured before neoadjuvant therapy. The first available value, 14.45 ng/mL, was obtained on 4 January 2026, 20 days after the single treatment cycle; a second value of 14.68 ng/mL was obtained during preoperative assessment, and the postoperative value was 6.08 ng/mL. The assay-specific reference interval was not preserved in the archived report; therefore, the manuscript no longer categorizes the two preoperative values as abnormal or elevated. In the absence of a pretreatment measurement and a verified reference interval, these values cannot establish a treatment-response or surveillance-biomarker relationship and are reported descriptively only.

Postoperatively, tislelizumab 200 mg every 3 weeks was selected as individualized maintenance therapy. The switch from pembrolizumab to tislelizumab was driven by the patient’s economic circumstances and treatment affordability, not by evidence of superiority or interchangeability. This non-standard, risk-adapted decision is not presented as a guideline recommendation. At the latest follow-up on 17 July 2026, approximately 6 months after surgery, follow-up assessments showed no evidence of recurrence or metastasis. The patient had experienced no maintenance-treatment-related adverse events and continued single-agent tislelizumab.

### 3.7. Biomarker Testing

PD-L1 combined positive score was not measured because the available diagnostic biopsy tissue was insufficient after routine pathological assessment. The multidisciplinary team did not delay treatment to obtain a repeat biopsy. Although PD-L1 CPS may enrich for benefit at a population level, it is not a deterministic individual predictor; nevertheless, the absence of CPS remains an important limitation [9]. AFP immunohistochemistry, SALL4, mismatch-repair proteins, microsatellite instability, Epstein–Barr virus, HER2, and next-generation sequencing were also unavailable, limiting mechanistic interpretation and treatment selection.

### 3.8. Patient Perspective

A separate written patient-perspective statement was not available. Written informed consent for publication of de-identified clinical information and accompanying images was obtained from the patient.

## 4. Discussion

This case is original because it combines synchronous ESCC and gastric cardia adenocarcinoma with regional hepatoid differentiation in one patient and documents their postoperative pathological findings after the same systemic exposure. The strongest lesson is diagnostic: a small biopsy can miss geographically restricted hepatoid differentiation, so extensive sampling and an appropriate immunohistochemical panel are important when morphology or clinical behavior is atypical [3,6,7,8].

The different postoperative regression scores should not be interpreted as proof of intrinsic biological resistance in the cardia lesion. Although both tumors were assigned cT2N0M0 at baseline, clinical staging does not fully capture tumor volume, viable-cell fraction, or microenvironmental differences. Histological lineage, anatomical site, drug sensitivity, sampling, and especially the administration of only one neoadjuvant cycle remain important confounders. Because both lesions were retrospectively reviewed with the same CAP modified Ryan system, the numerical scores are methodologically comparable; however, their biological meaning across two different primary tumors remains descriptive. Paired genomic or immune-microenvironment profiling would be required to attribute the difference to hepatoid biology.

The AFP interpretation was deliberately tempered. No true pretreatment AFP value was available, the assay-specific reference interval could not be retrieved, and the two preoperative measurements were obtained after treatment. The postoperative value of 6.08 ng/mL may reflect biological or analytical variation and does not validate AFP as a response or recurrence marker in this patient. AFP may still be measured during routine follow-up because hepatoid adenocarcinoma can be associated with AFP production, but any future change must be interpreted with repeated measurements, liver function, imaging, symptoms, and clinical context [8,20].

The neoadjuvant strategy was necessarily individualized. The pembrolizumab-taxane-platinum regimen was chosen primarily for the ESCC while providing systemic cytotoxic treatment for both lesions. MATTERHORN established perioperative durvalumab plus FLOT for resectable gastric or gastroesophageal-junction adenocarcinoma but did not include synchronous ESCC [13]. Thus, it did not provide a single evidence-based regimen for both primaries. Moreover, agranulocytosis prevented further treatment, and the patient proceeded to surgery after only one cycle. The cardia score of 3, therefore, cannot be interpreted as equivalent to poor regression after completion of a standard multi-cycle perioperative regimen.

Postoperative tislelizumab should be viewed as an individualized, non-standard decision. Evidence supporting adjuvant immune-checkpoint blockade in other perioperative settings cannot be directly extrapolated to this R0, ypN0 case after neoadjuvant chemoimmunotherapy. The switch from pembrolizumab was driven by affordability, not comparative efficacy. There is no evidence that switching PD-1 antibodies improves outcomes in this setting, and the current approximately 6-month follow-up is insufficient to evaluate benefit. The report therefore describes the decision transparently without recommending it as routine care.

Additional limitations include the limited lymph-node yield, absence of PD-L1 and molecular biomarkers, lack of retrievable high-power H&E fields and publication-quality gross or intraoperative photographs, and short follow-up. Only 10 nodes were examined, which may have led to nodal understaging and weakens confidence in ypN0. Although no recurrence, metastasis, or maintenance-related toxicity was identified by 17 July 2026, approximately 6 months of follow-up provides little prognostic information for an aggressive histology. Future reports should include standardized nodal dissection, paired molecular analysis, high-power morphology with a complete immunohistochemical panel, and longer surveillance. Multi-institutional registries may help clarify optimal management of similarly rare synchronous tumors.

## 5. Conclusions

Synchronous ESCC and gastric cardia adenocarcinoma with regional hepatoid differentiation is rare and diagnostically challenging. This case highlights biopsy under-sampling and the importance of separately staging and reassessing each lesion after systemic treatment. The CAP modified Ryan scores differed after one cycle of neoadjuvant chemoimmunotherapy, but the comparison remains descriptive because baseline burden, histology, treatment sensitivity, and abbreviated exposure are confounders. The available AFP measurements do not establish a surveillance biomarker, and the individualized postoperative PD-1 strategy cannot be evaluated with approximately 6 months of follow-up. Accumulation of molecularly annotated cases with longer observation is required.

## Figures and Tables

**Table 1:** Clinical timeline and key verified findings.

Date/Time Point	Clinical Event	Key Findings
Late 2025	Initial presentation and endoscopy	Progressive dysphagia; separate esophageal and gastric cardia lesions. Biopsies showed ESCC and cardia adenocarcinoma.
Late 2025	Baseline staging	PET/CT showed FDG-avid lesions without distant metastasis. AJCC 8th: ESCC cT2N0M0 (clinical stage II); gastric cardia adenocarcinoma cT2N0M0 (clinical stage I). The cardia epicenter did not involve the EGJ.
December 2025	Neoadjuvant treatment	One cycle of pembrolizumab 200 mg, nab-paclitaxel 300 mg, and cisplatin 100 mg was administered.
Second hospitalization/4 January 2026	Treatment-related adverse event and assessment	Agranulocytosis prevented a subsequent cycle. AFP was 14.45 ng/mL, 20 days after treatment; no pretreatment AFP value was available.
January 2026, preoperative	Recovery and restaging	After neutrophil recovery, endoscopy and CT showed improvement of the esophageal lesion but persistent cardia wall thickening; AFP was 14.68 ng/mL.
21 January 2026	Surgery	R0 resection of synchronous esophageal and cardia tumors with intrathoracic esophagogastric anastomosis and thoracic duct ligation.
January 2026	Postoperative pathology	CAP modified Ryan review: ESCC ypT1aN0M0, score 1; gastric cardia adenocarcinoma with approximately 30% hepatoid differentiation, ypT1aN0M0, score 3. Ten nodes were examined and were negative.
Postoperative follow-up	AFP assessment	AFP was 6.08 ng/mL. Because the assay-specific reference interval was not retrievable, values are reported numerically without labeling them abnormal.
17 July 2026	Maintenance therapy and latest follow-up	Tislelizumab 200 mg every 3 weeks was continued; the switch was based on affordability. At approximately 6 months after surgery, no recurrence, metastasis, or maintenance-related adverse event was identified.

## Data Availability

The original contributions presented in this case report are included in the article and Supplementary Materials. Further inquiries can be directed to the corresponding author, subject to institutional policy and patient privacy protection.

## Supplementary Materials

The following supporting information can be downloaded at: https://www.mdpi.com/article/10.3390/jcm15155979/s1, Supplementary File S1: CARE checklist; Supplementary Table S1: Pathological comparison of the two resected lesions after neoadjuvant treatment.

## References

[1] Ishikura H., Fukasawa Y., Ogasawara K., Natori T., Tsukada Y., Aizawa M. (1985). An AFP-producing gastric carcinoma with features of hepatic differentiation: A case report. Cancer.

[2] Nagai E., Ueyama T., Yao T., Tsuneyoshi M. (1993). Hepatoid adenocarcinoma of the stomach: A clinicopathologic and immunohistochemical analysis. Cancer.

[3] Su J.S., Chen Y.T., Wang R.C., Wu C.Y., Lee S.W., Lee T.Y. (2013). Clinicopathological characteristics in the differential diagnosis of hepatoid adenocarcinoma: A literature review. World J. Gastroenterol..

[4] Xia R., Zhou Y., Wang Y., Yuan J., Ma X. (2021). Hepatoid adenocarcinoma of the stomach: Current perspectives and new developments. Front. Oncol..

[5] Liu X., Cheng Y., Sheng W., Lu H., Xu X., Xu Y., Long Z., Zhu H., Wang Y. (2010). Analysis of clinicopathologic features and prognostic factors in hepatoid adenocarcinoma of the stomach. Am. J. Surg. Pathol..

[6] Osada M., Aishima S., Hirahashi M., Takizawa N., Takahashi S., Nakamura K., Tanaka M., Maehara Y., Takayanagi R., Oda Y. (2014). Combination of hepatocellular markers is useful for prognostication in gastric hepatoid adenocarcinoma. Hum. Pathol..

[7] Ushiku T., Shinozaki A., Shibahara J., Iwasaki Y., Tateishi Y., Funata N., Fukayama M. (2010). SALL4 represents fetal gut differentiation of gastric cancer and is diagnostically useful in distinguishing hepatoid gastric carcinoma from hepatocellular carcinoma. Am. J. Surg. Pathol..

[8] Wang B., Xie Y., Zheng L., Zheng X., Gao J., Liu X., Yuan Y., Li Z., Lu N., Xue L. (2021). Both the serum AFP test and AFP/GPC3/SALL4 immunohistochemistry are beneficial for predicting the prognosis of gastric adenocarcinoma. BMC Gastroenterol..

[9] Sun J.M., Shen L., Shah M.A., Enzinger P., Adenis A., Doi T., Kojima T., Metges J.-P., Li Z., Kim S.-B. (2021). Pembrolizumab plus chemotherapy versus chemotherapy alone for first-line treatment of advanced oesophageal cancer (KEYNOTE-590): A randomised, placebo-controlled, phase 3 study. Lancet.

[10] Janjigian Y.Y., Shitara K., Moehler M., Garrido M., Salman P., Shen L., Wyrwicz L., Yamaguchi K., Skoczylas T., Bragagnoli A.C. (2021). First-line nivolumab plus chemotherapy versus chemotherapy alone for advanced gastric, gastro-oesophageal junction, and oesophageal adenocarcinoma (CheckMate 649): A randomised, open-label, phase 3 trial. Lancet.

[11] Qiu M.Z., Oh D.Y., Kato K., Arkenau T., Tabernero J., Correa M.C., Zimina A.V., Bai Y., Shi J., Lee K.-W. (2024). Tislelizumab plus chemotherapy versus placebo plus chemotherapy as first-line treatment for advanced gastric or gastro-oesophageal junction adenocarcinoma: RATIONALE-305 randomised, double-blind, phase 3 trial. BMJ.

[12] Xu J., Kato K., Raymond E., Hubner R.A., Shu Y., Pan Y., Park S.R., Ping L., Jiang Y., Zhang J. (2023). Tislelizumab plus chemotherapy versus placebo plus chemotherapy as first-line treatment for advanced or metastatic oesophageal squamous cell carcinoma (RATIONALE-306): A global, randomised, placebo-controlled, phase 3 study. Lancet Oncol..

[13] Janjigian Y.Y., Al-Batran S.-E., Wainberg Z.A., Muro K., Molena D., Van Cutsem E., Hyung W.J., Wyrwicz L., Oh D.-Y., Omori T. (2025). Perioperative durvalumab in gastric and gastroesophageal junction cancer. N. Engl. J. Med..

[14] Sun Y., Chang W., Yao J., Liu H., Zhang X., Wang W., Zhao K. (2022). Effect of immune checkpoint inhibitors in patients with gastric hepatoid adenocarcinoma: A case report and literature review. J. Int. Med. Res..

[15] Zhou Y., Dong L., Dai L., Hu S., Sun Y., Wu Y., Pan T., Li X. (2023). Pathologic complete response of hepatoid adenocarcinoma of the stomach after chemo-immunotherapy: A rare case report and literature review. Front. Surg..

[16] Wang J., Zhan J., Rao Z., Su G., He Y., Zhou L., Wu J., Sun X., Xiang X. (2025). A multicenter retrospective study of PD-1 blockade plus chemotherapy as first-line therapy in advanced hepatoid adenocarcinoma of the stomach. Oncologist.

[17] Amin M.B., Edge S.B., Greene F.L., Byrd D.R., Brookland R.K., Washington M.K., Gershenwald J.E., Compton C.C., Hess K.R., Sullivan D.C. (2017). AJCC Cancer Staging Manual.

[18] College of American Pathologists Protocol for the Examination of Specimens from Patients with Carcinoma of the Esophagus, Version 4.2.0.1. https://documents.cap.org/protocols/Esophagus_4.2.0.1.REL_CAPCP.pdf.

[19] College of American Pathologists Protocol for the Examination of Specimens from Patients with Carcinoma of the Stomach, Version 4.3.0.0. https://documents.cap.org/protocols/Stomach_4.3.0.0.REL_CAPCP.pdf.

[20] Wang Y.K., Shen L., Jiao X., Zhang X.T. (2018). Predictive and prognostic value of serum AFP level and its dynamic changes in advanced gastric cancer patients with elevated serum AFP. World J. Gastroenterol..

