# Supplementary material for: Case Report: Synchronous Esophageal Squamous Cell Carcinoma and Gastric Cardia Adenocarcinoma with Hepatoid Differentiation After Neoadjuvant Chemoimmunotherapy
Source: J Clin Med. 2026 Jul 31;15(15):5979. doi: 10.3390/jcm15155979 (PMC13466451; doi:10.3390/jcm15155979)
Supplement: Supplementary file 1 [file jcm-15-05979-s001.zip › Supplementary File S1-CARE_Checklist.pdf]

CARE Checklist for Case Reports

Manuscript: Case Report: Synchronous Esophageal Squamous Cell Carcinoma and Gastric Cardia Adenocarcinoma with Hepatoid Differentiation Showing Heterogeneous Pathological Responses to Neoadjuvant Chemoimmunotherapy

Journal: Journal of Clinical Medicine

Completed for submission as supplementary material. Page numbers refer to the rendered Word manuscript prepared in the JCM template and should be rechecked against the final PDF generated by the submission system.

Topic	Item	CARE checklist item	Reported in manuscript	Page(s)
Title	1	The diagnosis or intervention of primary focus followed by “case report”.	Title page	1
Keywords	2	Two to five key words that identify diagnoses or interventions, including “case report”.	Title page	1
Abstract	3a	Introduction: what is unique about this case and what does it add?	Abstract	1
Abstract	3b	The patient’s main concerns and important clinical findings.	Abstract	1
Abstract	3c	The main diagnoses, therapeutic interventions and outcomes.	Abstract	1
Abstract	3d	Conclusion: main take-away lessons.	Abstract	1
Introduction	4	Brief summary of background and rationale with relevant literature.	Introduction	2
Patient Information	5a	De-identified demographic information and main concerns/symptoms.	Patient Information	3
Patient Information	5b	Medical, family and psychosocial history, including relevant genetic information when available.	Patient Information	3
Patient Information	5c	Relevant past interventions and outcomes.	Patient Information	3
Clinical Findings	6	Relevant physical examination and clinical findings.	Patient Information / Clinical Findings	3
Timeline	7	Important dates and times in the case.	Table 1 and Figure 1	3, 9
Diagnostic Assessment	8a	Diagnostic methods, including physical examination, laboratory testing, imaging, pathology and other evaluations.	Diagnostic Assessment	4
Diagnostic Assessment	8b	Diagnostic challenges, including financial, language or cultural issues where relevant.	Diagnostic Assessment / Discussion	4, 6-8
Diagnostic Assessment	8c	Diagnostic reasoning including differential diagnoses.	Diagnostic Assessment	4
Diagnostic Assessment	8d	Prognostic characteristics where applicable.	Diagnostic Assessment / Postoperative Pathology	4-6
Therapeutic Intervention	9a	Types of intervention, including pharmacologic, surgical, preventive and self-care interventions.	Therapeutic Intervention	5
Therapeutic Intervention	9b	Administration of intervention, including dosage, strength and duration.	Therapeutic Intervention / Follow-up	5-6

Therapeutic Intervention	9c	Changes in intervention and reasons.	Follow-up and Outcomes	6
Follow-up and Outcomes	10a	Clinician- and patient-assessed outcomes.	Follow-up and Outcomes	6
Follow-up and Outcomes	10b	Important follow-up diagnostic and other test results.	Follow-up and Outcomes	6
Follow-up and Outcomes	10c	Intervention adherence and tolerability.	Therapeutic Intervention / Follow-up	5-6
Follow-up and Outcomes	10d	Adverse and unanticipated events.	Therapeutic Intervention	5
Discussion	11a	Strengths and limitations of the case report.	Discussion	6-8
Discussion	11b	Relevant medical literature.	Discussion	6-8
Discussion	11c	Rationale for conclusions.	Discussion	6-8
Discussion	11d	Primary take-away lessons.	Discussion / Conclusions	6-8
Patient Perspective	12	Patient perspective or experience where possible.	Patient Perspective	6
Informed Consent	13	Patient informed consent.	Informed Consent Statement	14
