# Supplementary material for: Case Report: Synchronous Esophageal Squamous Cell Carcinoma and Gastric Cardia Adenocarcinoma with Hepatoid Differentiation After Neoadjuvant Chemoimmunotherapy
Source: J Clin Med. 2026 Jul 31;15(15):5979. doi: 10.3390/jcm15155979 (PMC13466451; doi:10.3390/jcm15155979)
Supplement: Supplementary file 1 [file jcm-15-05979-s001.zip › Supplementary Table S1.pdf]

Supplementary Table S1. Pathological comparison of the two resected lesions after neoadjuvant treatment.

Feature	Lesion 1: esophageal lesion	Lesion 2: gastric cardia lesion
Baseline AJCC 8th stage	cT2N0M0, clinical stage II	cT2N0M0, clinical stage I (gastric staging; epicenter did not involve the EGJ)
Gross findings	2 cm × 2 cm rough mucosal area in a 9-cm esophageal segment	1.5 cm × 2 cm × 0.5 cm gray-white friable mass on the lesser-curvature side of the cardia
Histology after treatment	High-grade squamous intraepithelial neoplasia with focal carcinoma	Grade II-III adenocarcinoma, Lauren mixed intestinal-diffuse type (~70%), with regional hepatoid adenocarcinoma (~30%)
Depth of invasion	Lamina propria	Muscularis mucosae
Post-treatment stage	ypT1aN0M0	ypT1aN0M0
Tumor regression method	CAP modified Ryan four-tier system	CAP modified Ryan four-tier system
Tumor regression score	Score 1 (near-complete response)	Score 3 (poor or no response)
Lymphovascular/perineural invasion	Not identified	Not identified
Immunohistochemistry	Consistent with squamous differentiation based on morphology	AE1/AE3 positive; CDX2 partially positive; Glypican-3 regionally positive; HepPar-1/Hep-1 regionally positive; CEA, synaptophysin, and P40 negative
Margins	Circumferential margin negative	Proximal and distal margins negative
Lymph nodes	Paraesophageal lymph nodes: 0/5	Cardia lymph nodes: 0/5; left gastric soft tissue negative
Interpretation	Score 1 after one neoadjuvant cycle	Score 3 after one neoadjuvant cycle; the cross-lesion comparison remains descriptive because of different histology, tumor burden, and treatment sensitivity
